# The experience of agency in human-computer interactions: a review

**DOI:** 10.3389/fnhum.2014.00643

**Published:** 2014-08-21

**Authors:** Hannah Limerick, David Coyle, James W. Moore

**Affiliations:** ^1^Department of Computer Science, Bristol Interaction and Graphics, University of BristolBristol, UK; ^2^Department of Psychology, Goldsmiths, University of LondonLondon, UK; ^3^School of Experimental Psychology, University of BristolBristol, UK

**Keywords:** sense of agency, human computer interaction, control, technology, computer assistance, joint action

## Abstract

The sense of agency is the experience of controlling both one’s body and the external environment. Although the sense of agency has been studied extensively, there is a paucity of studies in applied “real-life” situations. One applied domain that seems highly relevant is human-computer-interaction (HCI), as an increasing number of our everyday agentive interactions involve technology. Indeed, HCI has long recognized the feeling of control as a key factor in how people experience interactions with technology. The aim of this review is to summarize and examine the possible links between sense of agency and understanding control in HCI. We explore the overlap between HCI and sense of agency for computer input modalities and system feedback, computer assistance, and joint actions between humans and computers. An overarching consideration is how agency research can inform HCI and vice versa. Finally, we discuss the potential ethical implications of personal responsibility in an ever-increasing society of technology users and intelligent machine interfaces.

## Introduction

The sense of agency is the experience of controlling both one’s body and the external environment. This experience has received a considerable amount of attention in the field of cognitive neuroscience, due in part to the recognition that a disordered sense of agency is central to illnesses such as schizophrenia (Frith, 1992). The sense of agency is also an important part of human consciousness more generally, forming a fundamental aspect of self-awareness (Gallagher, 2002). In this review, we will primarily focus on the sense of agency for control over the external environment. This is because it is most pertinent to the human-computer-interaction (HCI) issues we consider.

The sense of agency is a vital consideration for assessing how people experience interactions with technology, a core focus for research in the field of HCI. The seventh of Shneiderman’s Rules of Interface Design states that designers should strive to create computer interfaces that *“support an internal locus of control”* (Shneiderman and Plaisant, 2004). This is based on the observation that users *“strongly desire the sense that they are in charge of the system and that the system responds to their actions”*. What makes our understanding of agency in HCI especially pertinent is the fact that an increasing number of our everyday agentive interactions involve technology. During interactions with technology, the simple process of producing an action to cause an intended outcome is endowed with a whole host of possible variables that can alter the agentive experience dramatically. Thus both cognitive neuroscience and HCI seek to understand how humans experience agency and control over action execution. The aim of this review is to examine the links between sense of agency in cognitive neuroscience and HCI and highlight some possible new research directions.

We pose that an interdisciplinary combination of HCI research and cognitive neuroscience to investigate the sense of agency can provide a rich and promising new research area that has the potential to inform both fields in novel ways. Research into the sense of agency stands to benefit from the new interaction techniques rapidly being developed in the field of HCI such as gestural input, physiological or intelligent interfaces and assistance methods. Thus enabling novel ways of producing actions to be incorporated into such research. Moreover, testing agency in more “real-world” settings can lead to new insights regarding the nature and parameters of agentive experiences in everyday interactions. Equally, HCI research can take advantage of the relative maturity of neurocognitive research and the reliable metrics for the experience of volitional control that have been developed. An incorporation of such metrics will encourage the HCI researcher to consider the sense of agency as a quantifiable experience in future research. Furthermore, understanding the neurocognitive processes and mechanisms that support this experience provides an important evidence base and guide for interface design. The first section of this paper briefly considers the theoretical and methodological background of research on the sense of agency. We then discuss the potential implications and areas of overlap of these theories and methods for three specific areas of HCI research: (1) input modalities and system feedback; (2) computer assistance; and (3) collaboration and attribution of agency.

## Theoretical and methodological background into the sense of agency

As stated above, the sense of agency is the experience of controlling both one’s body and the external environment. On this definition, control is central to the experience of being an agent. In the psychological literature a number of studies have investigated the relationship between control and agency. For example, it has been shown that sense of agency is altered by a manipulation of the statistical relationship between actions and effects (Moore et al., 2009) and by a manipulation of the perception of control over action (Desantis et al., 2011). More recent work by Kumar and Srinivasan (2014) has also looked at how sense of agency is influenced by control specified at different hierarchical levels. This work shows, in part, that when higher-level control is exercised (i.e., goal-level control) lower level control processes (i.e., perceptuo-motor control) have no influence on sense of agency. This relationship between control and sense of agency is highly relevant in the context of HCI, given the fact that different HCI applications involve different kinds of control manipulations.

A phenomenological distinction has been made between the “Feeling of Agency” and the “Judgement of Agency” (Synofzik et al., 2008). The feeling of agency refers to the implicit, pre-reflective, low-level feeling of being the agent of an action. The judgement of agency describes the explicit judgement and attribution of agency to oneself or another on a conceptual level. Traditionally there are two theoretical views regarding the neurocognitive processes underlying the sense of agency. Some have suggested that the sense of agency arises principally from internal processes serving motor control (Blakemore et al., 2002; Haggard, 2005). On the other hand, external situational cues have been emphasized (Wegner, 2002, 2003). However, it is now becoming increasingly recognized that this is a false dichotomy and that various cues contribute to the sense of agency (Wegner and Sparrow, 2004; Wegner et al., 2004; Moore et al., 2009; Moore and Fletcher, 2012; Kranick and Hallett, 2013). These cues include internal sensorimotor signals and external situational information (Moore and Fletcher, 2012). Moreover, it has been suggested that the influence of these cues depends on their reliability (Moore and Fletcher, 2012), implying some form of optimal cue integration. According to the cue integration concept, multiple agency cues are weighted by their relative reliability and then optimally integrated to reduce the variability of the estimated origins of an action.

**Figure 1:**
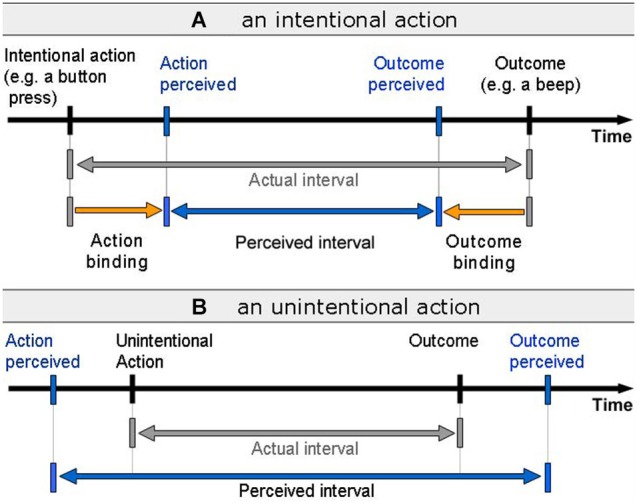
**Intentional Binding. (A)** During voluntary action, the perceived time of action and outcome are shifted toward one another, resulting in a shorter perceived interval between action and outcome. **(B)** During involuntary actions, the perceived time interval between action and outcome is longer than the actual delay.

Researchers have developed numerous ways of measuring the components of the sense of agency experimentally. The explicit judgement of agency is typically measured by verbal report by asking participants to rate their feeling of agency during a task or simply state whether they were the agent or not. Measures have also been developed to probe implicit aspects of sense of agency. These include sensory attenuation paradigms (e.g., Blakemore et al., 1998) and intentional binding (e.g., Haggard et al., 2002). In this review we focus primarily on intentional binding. In this paradigm participants report the perceived time of voluntary action initiation and the consequent effects using the so-called Libet clock. Haggard et al. (2002) found that when participants caused an action, their perceived time of initiation and the perceived time of the outcome where brought closer together, i.e., the perceived interval between voluntary actions and outcomes was shorter than the actual interval (Figure 1). In the case of involuntary actions the perceived interval was found to be longer than the actual interval. This phenomenon is known as “intentional binding”, and is seen as an implicit metric for the sense agency.

Intentional binding is a widely used implicit measure of the sense agency and the effect has been replicated widely and in a number of settings (e.g., see Wohlschläger et al., 2003; Engbert and Wohlschläger, 2007; Moore et al., 2009; Aarts and van den Bos, 2011). More recently, alternative intentional binding measures have been developed, such as the direct interval estimation procedure where the participant is required to estimate the interval between actions and outcomes (e.g., see Moore et al., 2009; Humphreys and Buehner, 2009; Coyle et al., 2012). As stated above, intentional binding is the predominant measure considered in this review. The main reason for this is that while it seems particularly well suited to the nature of agent interaction during many interactions with technology, it has not yet been widely applied in the HCI domain. One key advantage of intentional binding in the context of HCI is that it is typically measured at sub-second sensorimotor timescales, which are common in HCI. An additional benefit of intentional binding in the context of HCI is that it offers a measure of the *degree* of sense of agency the individual experiences, rather than being a binary “me” vs. “not me” measure. However, it is important to note that intentional binding may not be best suited for assessing sense of agency for all types of tasks within HCI, such as those agentive interactions operating at much longer timescales (although see Faro et al., 2013, for review of literature suggesting that binding may operate at longer timescales).

## Input modalities and system feedback

The first point of contact between the sense of agency and HCI we wish to consider is the importance of input modalities. Input modalities are the sensors or devices by which the computer receives input from the human, e.g., a keyboard or mouse. The input modality is central to the process of producing actions in order to bring about the user’s desired state changes in the computer and thus also central to the sense of agency over the action. HCI research is interested in how to optimally turn psychological states (such as intentions) into state changes within the computer. The user’s intentions and the system’s state differ considerably in form and content and one of the challenges of HCI is to bridge this gap. This separation is known as the Gulf of Execution (Norman, 1986). The input modality of the system is central to bridging the Gulf of Execution. Norman (1986) states:

*“Execution of an action means to do something, whether it is just to say something or perform a complex motor sequence. Just what physical actions are required is determined by the choice of input devices on the system, and this can make a major difference in the usability of the system. Because some physical actions are more difficult than others, the choice of input devices can affect the selection of actions which in turn affects how well the system matches with intentions”*.

More recently a similar message has been emphasized by Williamson et al. (2009) who state:

*“A computer interface facilitates control. It provides a set of mechanisms by which a human can drive the belief of a system about a user’s intentions towards a desired state over a period of time. Control requires both display to the user and input from the user; computers feedback state to a user, who modifies his or her actions to bring about the required change of state”*.

**Figure 2:**
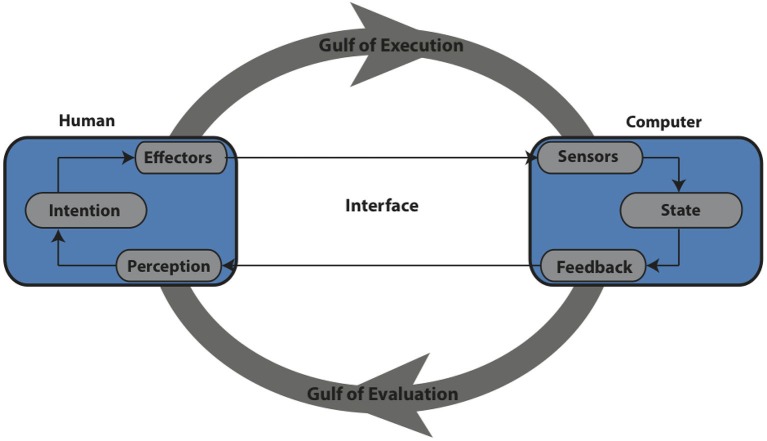
**A closed loop input system, based on Norman (1986) and Williamson et al. (2009)**.

In recent years HCI researchers have developed a wide range of new interaction techniques and devices such as speech and gestural control. These are rapidly becoming common place, with everyday devices having the option of being controlled by such interaction including, smart phones (Apple’s Siri), televisions (Samsung’s Smart TV), computers (Leap Motion) and games consoles (Microsoft Kinect). Each new method of controlling technology presents new challenges to HCI researchers. New input modalities offer different ways of “bridging the Gulf of Execution” including distinct action initiation requirements, feedback mechanisms, and device capabilities (Figure 2). This has the potential to dramatically reshape the experience of control and agency. In this section we consider some of the ways in which agency research can help to inform such issues. From a cognitive neuroscience perspective different modes of action execution pose interesting questions. Experimental investigations into the sense of agency have typically involved participants controlling their environment via conventional input devices such as a keyboard or mouse. Altering the sensorimotor requirements for action execution presents an opportunity to further investigate the sense of agency during distinctly new sensorimotor requirements.

### Input modalities

**Figure 3:**
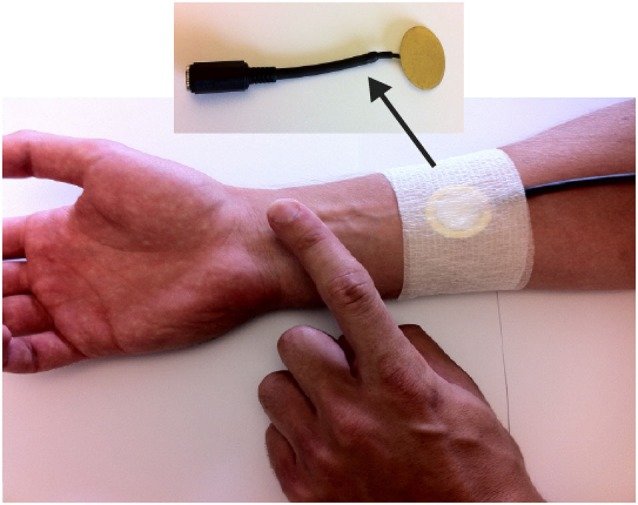
**In Coyle et al. (2012) a piezo-electric contact microphone was placed on the user’s arm and connected to a computer**. Vibrations on the user’s arm were monitored to detect when the user tapped on their arm.

To begin addressing the impact of input modalities on the sense of agency, Coyle et al. (2012) conducted an experiment employing intentional binding as an implicit measure of users’ sense of agency for two distinctive input techniques. In one condition participants pressed a button on a keyboard to cause an outcome (a beep). In the second condition participants wore a skin-based input device and tapped their arm to cause a beep (Figure 3). Results show that intentional binding was significantly greater for skin-based input than the keyboard input, thus indicating a stronger sense of agency with skin-based input. From an input design perspective this is interesting as it indicates that skin-based input is experienced as significantly more responsive than button-based input. More broadly, Coyle et al. provided evidence that different interaction techniques can provide different experiences of agency to those offered by traditional mouse or keyboard interactions. It also demonstrated the potential of intentional binding to quantify this difference. In future this method can be used to assess and quantify the differences for a larger range of interaction techniques, including changes more subtle than those assessed here. We could for example assess the difference in the experience of agency during interactions with a touch screen via a stylus vs. direct finger interaction, or differences in interactions that incorporate techniques such as haptic, embodied or physiological input. We can also compare the different interaction experiences for a specific input technique when other conditions of the interactions change, e.g., when conditions such as system feedback, reliability or latency are varied. Greater consideration is given to these possibilities below.

The finding that skin-based input results in greater intentional binding also raises interesting questions regarding the underlying cognitive processes for the sense of agency. One possible explanation for the higher sense of implicit agency measured in the skin-based input is that, with a self directed, skin-based action, there is a higher degree of congruence between the internally predicted sensory output of the action and the actual sensory output of the action. Intentional binding may be strengthened when the individual is more sensorially aware of their action. Another possible explanation in line with the cue integration theory for agency is that for the skin-input conditions, participants receive additional sensory agency cues from the passive limb, which is acting as the input modality. This may serve to increase sense of agency. A final possible explanation is linked to the finding that actions aimed at the self are associated with increased activity within the motor system (Master and Tremblay, 2010). Given that the sense of agency is closely tied to sensorimotor processes, increased activity within this system might increase the sense of agency. These are all possibilities that we are currently exploring. Whatever the explanation, this study shows how the new modes of interaction being developed in HCI can open up new avenues of enquiry for the neurocognitive understanding of the sense of agency.

### Reliability

Coyle et al. (2012) represents an early application of intentional binding to explicitly address an HCI research question. But there are many more specific input design questions and trade offs that can be informed by their approach. System reliability is one such issue.

Many input techniques suffer from varying degrees of reliability, due to the fact that the interaction requires the computer’s sensors to recognize and then classify the intention of the user, which is not always clear-cut and often noisy. Consider for example a speech interface and the various possible accents the user may have. A speech system designed to accommodate many different accents is likely to result in more incorrect classifications of peoples’ utterances. Speech systems could be made more reliable through initial training periods or by allowing the system more time to classify utterances. But this reduces the responsiveness of the speech input system. In a similar vein a gesture recognition system like the Microsoft Kinect is required to recognize a wide range of mid-air gestures. Even for simple gestures there are variations in the way different people will execute the gesture. A system that allows leeway for variations in action execution will be more flexible, but again may result in more misclassifications of peoples’ actions. Designers of such systems are therefore required to make trade-offs between constraints such as accuracy and flexibility, both of which affect system reliability.

Reliability is analogous to the predictability of an action and has been found in neurocognitive research to affect sense of agency. Empirical evidence suggests that participants experience a lower sense of agency for unexpected outcomes of their actions (Sato and Yasuda, 2005). Moore and Haggard (2008) investigated inference and prediction for conditions where there was either a high or low probability of an outcome. The results indicated that in both probability conditions, participants exhibited binding for situations where the action was followed by the outcome. For high probability conditions, participants also exhibited intentional binding for trials where the action was not followed by an outcome, suggesting that a strong prediction was sufficient to generate the binding effect. With regard to input modalities and reliability, these results suggest that the more reliable an input method is, both in terms of matching the intended outcome and in predictability, the greater the sense of agency experienced by the user. In future similar approaches may provide HCI researchers with a concrete means of investigating how reliable a system needs to be before people begin to experience significant reductions in their sense of agency. Evidence from such studies will help designers to make more informed decisions regarding the reliability trade-offs in new input systems.

### System feedback

In addition to the input modality, control over a computer system requires feedback to inform the user of the system’s current state, the actions required to bring about changes in the system’s state in line with their intentions and the success of those actions. The user can then use this feedback to modify their consequent actions to bring about the next desired outcome. In HCI, the mode of feedback and the information the interface provides regarding the system’s state is again an important consideration. Parallel to the Gulf of Execution, Norman describes the Gulf of Evaluation (Norman, 1988), which refers to the mismatch between the system’s feedback regarding it’s actual state and how this state is perceived by the user in terms of their expectations and intentions (see Figure 2). The Gulf of Evaluation will differ depending on the particular interface, context, requirements and user expectations. For example, a mobile phone interface and an automatic flight deck will have distinctly different Gulfs of Evaluation and therefore require different forms of feedback to be presented to the user.

Typically, when interacting with technology users make an action and then receive sensory feedback about their action. Consistency between predicted sensory feedback and actual sensory feedback during action execution has been the focus of several studies in cognitive neuroscience. Interestingly, empirical evidence indicates that the sense of agency is malleable and feedback can be distorted to lead participants to misattribute their own actions as being caused by another agent or visa versa. In cases where the outcome of an action is incongruent with participants’ predicted sensory outcome, agency can be misattributed to an external source (Sato and Yasuda, 2005). Conversely Sato and Yasuda (2005) also induced a false sense of agency for an externally generated action that matched participants’ predictions. Farrer et al. (2008) found that deviations in the visual feedback of a moving curser associated with joystick movement beyond 50° led participants to explicitly attribute their movements to another agent irrespective of their implicit sensorimotor movements. System feedback presented to the user may also be in the form of contextual information. Such feedback might have a profound effect on the user’s experience of agency. For example, Desantis et al. (2011) demonstrated that prior causal beliefs about the agent of an action led participants to experience less implicit sense of agency for self-generated actions that they believed to be caused by another agent.

In order to achieve optimal control over an interface it will be beneficial to the interface designer to understand how sensory feedback of the interface modulates the sense of agency in various contexts. Evidence regarding the degree to which sensory feedback should match the user’s predicted feedback is valuable for developing effective input modalities. This is especially so, considering the evidence that mismatches between predicted outcome and actual outcome can actually lead to misattributed sense of agency.

### Latency

Another factor to note when considering input modalities and the sense of agency is the latency imposed between the action and it’s consequent outcome. Latency is commonly presented as an issue in HCI due to technological constraints within the system. This can interfere with perceptual constraints such as attention span or memory load. Therefore another key question in HCI research is how best to overcome latency in a way that suits the user’s perceptual capacities. An example would be the Roto and Oulasvirta (2005) early work on web browsing on a mobile phone. They identified several temporal constraints, including the speed of the network connection, the phone’s processing abilities, and the user’s visual attention span. They found that a user’s attention typically shifts away from a screen after 4–8 s. At the time of their research, mobile web browsing suffered from page download times being over 5 s. Roto and Oulasvirta suggested that a solution to this is multimodal feedback, with tactile feedback (vibration) helping to reduce the need for visual attention beyond which is natural.

In a similar vein to the web-browsing example, neurocognitive experimental techniques have the potential to validate design decisions regarding latencies in a range of contexts. Empirical evidence indicates that the intentional binding phenomenon breaks down beyond 650 ms for a simple button-pressing task (Haggard et al., 2002). However, there is also evidence for intentional binding still being intact at 2250 ms for a conflict resolution task (Berberian et al., 2012). In order to optimally overcome the effect that latency has on control, an understanding of how the sense of agency behaves and is modulated over time-scales is important. In many cases decisions on latency will also involve trade-offs regarding system feedback and system reliability. For example, an input classification system can be made more reliable by allowing it more time to make an accurate classification of peoples’ actions, but this will increase the latency of the system.

### Brain machine interfaces

We conclude this section on input modalities and system feedback by considering one final input technique that has relevance for all of the issues we have discussed above. Brain Machine Interfaces (BMI) use different aspects of the brain’s cortical activity such as P300 (Farwell and Donchin, 1988) or slow cortical potentials (Hinterberger et al., 2004) to control objects such as prosthetic arms (Velliste et al., 2008), external devices (Wolpaw and McFarland, 2004) and computer cursers (Kennedy et al., 2000). BMI suffers from variable reliability largely due to the fact that it is EEG based and therefore the bandwidths involved are slow, noisy and suffers variable delays between action and outcome (Williamson et al., 2009). The field of HCI is currently attempting to improve control in BMI to make it more effective in therapeutic contexts. One aspect of this is faster command execution (Minnery and Fine, 2009). Neurocognitive research into the sense of agency may offer insights into ways to modify agency under such latencies. Metrics used to measure the sense of agency can also provide guidance regarding optimum time delays a system can take to respond to an action, beyond which agency starts to break down. BMI has broad feedback channels with the possibility of providing the user with rich sensory feedback (Williamson et al., 2009) and thus these feedback channels could be exploited to provide sensory cues or external contextual cues which increase the experience of agency despite the latencies involved.

## Computer assistance

Computer systems that assist us in completing tasks are increasingly common and are likely to become ever more common-place given the increasing capabilities of technology and with the development of a broader range of intelligent machine interfaces. The degree to which computers assist us can vary from “high-assistance”, such as fully automatic flight decks, to “low assistance”, such as the smoothing or snap to point techniques that are used to make pointing with a mouse on a desktop computer more accurate. The manner in which the computer “assistant” is presented can also vary considerably and be made more or less explicit to the human user. Terveen (1995) identified two broad forms of computer assistance. The first is human emulation, where a computer assistant is endowed with human like abilities and an anthropomorphic representation, which is designed to ultimately mirror human-human interaction. We consider this form of assistance in Section Collaboration and Attribution of Agency below. In the present section we consider Terveen’s second category of computer-assisted action. Here the computer assistance is not presented in an anthropomorphic form and the assistance is not always made explicit to the user. The aim of such systems is generally to combine both the human and computer’s unique abilities to more effectively achieve a particular goal.

Intelligent interfaces and computer-assisted actions are interesting for many reasons, not least because of the varying degrees of control given to the user and the potential to introduce a grey area between voluntary and involuntary action. HCI research is interested in the many interactions now occurring in this grey area. For example, what happens to a person’s sense of agency when they voluntarily initiate an action, but a computer then steps in to complete the action? This agentive ambiguity in interactions with intelligent technologies also presents interesting challenges for research into the sense of agency.

### Task automation

Many tasks are now automated by computers and machines, requiring the user to simply monitor the activity and intervene when required. Some automated tasks are already common in everyday life, e.g., aircraft control and factory automation. Other examples, which once seemed like science fiction, are now commonplace in research settings and close to becoming an everyday occurrence, including self-driving cars and robotic surgery. In developing such systems designers need to think carefully about the optimal balance between computer assistance and human sense of agency. This is particularly important in safety critical systems and in semi-automated systems where a human supervising the task is held responsible for task failures.

Berberian et al. (2012) investigated the participants’ sense of agency when performing the complex task of flying a plane using a flight simulator under different levels of automation. The task required the participant to observe a flight plan and after a random time interval, a conflict occurred due to the presence of another plane. The participant was required to decide an appropriate command and implement it using a button-based interface. The action was followed by visual and auditory feedback informing the user whether they were successful in their conflict resolution. Participants were asked to estimate the time interval between the keypress and the auditory feedback. There were varying levels of automation of the task, from the user having complete control (no automation) to the computer executing the entire task with the participant simply observing (full automation). Berberian et al. found that with increasing levels of automation the participant’s estimate of action-outcome time interval duration increased—indicating that more assistance leads to less implicit sense of agency during the task. The authors concluded that the intentional binding measure of agency is a promising metric for HCI research and can assist in the optimal development of such operator control interfaces.

### Computer assisted movements

A vast majority of our interactions with computers require us to make motor actions. Therefore interface designers have focused efforts into optimally developing interfaces to compliment the dynamics of human motor actions. Coyle et al. (2012) investigated how the sense of agency is effected when a computer assists user’s mouse movements. Participants undertook a task in which they were required to point and click on an onscreen target using a standard mouse. During the experiment the computer gave participants different levels of targeting assistance in achieving this task. Once the participant clicked the target, an auditory tone occurred after a random interval. Implicit sense of agency was measured during this task by incorporating an interval estimation based intentional binding measure between clicking the target and hearing the tone. The results indicated that, although participants were aware of the varying assistance levels, at mild level of computer assistance they still experienced intentional binding, suggesting an implicit level of agency occurring for the action. The intentional binding measure for two further assistance levels (medium and high assistance) indicated a significant loss of agency. This suggests that, when interacting with a computer via assisted mouse movements, there is a point up to which users can be assisted and still feel a sense of agency, however beyond this point the experience of agency breaks down, even in situations where the computer correctly executes the human’s intentions.

Similarly, Kumar and Srinivasan (2013) ran an experiment in which participants were asked to click targets on a screen, using a joystick and trigger button. They manipulated the level of control provided to the user at the sensorimotor level–and measured the impact control has over implicit (intentional binding) and explicit (verbal report) sense of agency. The level of control provided to the joystick movements was varied from low, medium and full control. Once the trigger button was pressed (action), a blue circle would appear on the screen (outcome) and participants were asked to estimate the action-outcome interval. Participants also verbally reported their sense of authorship for the action. For tasks where the participants were unsuccessful in hitting the target and thus not achieving high-level goal, the results are consistent with that of Coyle et al. (2012) and Berberian et al. (2012) where intentional binding decreases as a function of automaticity provided during the task. However, when the goal was achieved, intentional binding did not show the same pattern.

The investigations above highlight that the sense of agency may be a graded experience in situations where the line between voluntary and assisted action is gradually blurred. We suggest that metrics for the sense of agency applied in the development of assisted control tasks would allow the interface designer to address the point where the experience of agency becomes disrupted. With regard to the cognitive basis for the sense of agency, the finding that there is a graded loss of sense of agency with increasing assistance is potentially consistent with our current understanding of sensorimotor prediction. For example, increasing assistance may result in internal sensorimotor predictive models becoming less accurate at predicting the next sensory state; this could therefore give rise to reduced congruence between the predicted sensory state and that actual sensory state. Therefore resulting in a reduced sense of agency for the action, of course, this requires further investigation.

## Collaboration and attribution of agency

Finally we turn to more explicit forms of computer assistance and the subject of human emulation. Here a computer agent is endowed with human like abilities and often an anthropomorphic representation that is designed to ultimately mirror human-human interaction (Terveen, 1995). We explore the relevance of the perceptual representation of a computer agent and the impact this has on the sense of agency when collaborating with computer agents as co-actors to achieve a shared goal. We also consider the process of attributing agency to a computer agent and how research into the sense of agency may help inform these areas within HCI and joint action.

The question of collaboration and attributed agency is also particularly relevant to the branch of HCI that focuses on humans’ interaction with robots—Human Robot Interaction (HRI). Robotics has made significant advances and is progressing to integrate robot entities into people’s everyday lives (Murphy et al., 2010). Effective and optimal implementation of human-robot collaboration techniques rely on HCI research to provide an understanding of the cognitive mechanisms involved in representing, understanding and communicating shared intentions between a human and a computer. HRI is faced with the same challenges in reciprocally representing and communicating human intentions and the system state of the robot. We believe the sense of agency and how we attribute it is an important cognitive consideration for HRI research when assessing how humans relate to robot co-actors.

The sense of agency is also an important consideration for the design of embodied virtual agents. Embodied agents are virtual humans that can engage with people in a human like manner and aim to both understand and generate speech, gestures and facial expressions (Cassell, 2000). Cassell states: *“they are a type of software agent insofar as they exist to do the bidding of their human users, or to represent their human users in a computational environment”*. Such agents have been investigated in application areas including education (Cassell, 2004; Ogan et al., 2012), healthcare (Bickmore and Gruber, 2010) and entertainment (Lim and Reeves, 2010).

The relevance of this section extends to cognitive science research. A burgeoning area of research on sense of agency investigates it in social settings (Sebanz et al., 2006; Sebanz, 2007; Pacherie, 2013). One key area of interest is how social context modifies the individual’s sense of agency. A number of important consequences of social context have been identified (Pacherie, 2013). One of these is a quantitative effect: social context can alter the strength of sense of agency. For example, in a sensorimotor learning study by van der Wel et al. (2012), they found little difference in sense of agency between participants acting along and with another at the beginning of the task. However as participants became more acquainted with the task significant differences emerged, with the joint action setting associated with a weaker sense of agency.

The majority of this work has so far focused on joint action between human agents. However, joint action between human and *computer* agents is now an important consideration both for agency and HCI research. In the following section we explore this issue in more detail.

### Computers vs. human co-actors

One significant consideration is how our sense of agency for actions differ when collaborating with computer vs. human partners. A study by Obhi and Hall (2011) addressed this idea by measuring intentional binding in a joint action task with both a computer partner and a human partner. The findings suggest that humans implicitly consider human-human joint actions very differently to human-computer joint actions. The task involved a participant acting with either a hidden human or computer partner making silent key-presses from behind a screen to cause a tone. Explicit information regarding who was actually responsible for the tone was given. Intentional binding measures were recorded for the action-outcome interval along with the subject’s explicit beliefs about who caused the tone. The results showed that the intentional binding was present when paired with another human, regardless of whether they explicitly knew they were the agent or not. This suggests an implicit sense of agency for their co-actor’s actions and thus indicating a “we-agency” for the action. For trials with the computer, no intentional binding occurred, even for trials where participants explicitly knew that they were the agent. These findings are compelling because they suggest that implicit agency for self-generated actions are overridden or inhibited when the participant is aware that a computer is the co-actor. The authors speculate that the breakdown in implicit agency here suggests that participants subconsciously develop a belief that when paired with a computer they have no control over the task. Furthermore, they suggest that the criteria for forming “we-agency” are based on comprehending other’s intentions in a similar way to one’s own, which may be more difficult with computers.

A similar investigation by Wohlschläger et al. (2003) found analogous effects. They measured the perceived onset time of self, other-human and machine generated actions. The results indicated that the participants had a delayed awareness of action for the self and other-human conditions. However, participants had an anticipatory awareness of actions in the machine conditions. In a second experiment Wohlschläger et al. (2003) controlled for the fact that during the machine-action condition there was a lack of visual information corresponding to the hand movement seen in the self and other-human conditions. Therefore, the study was repeated using a rubber hand for the machine-action trials. This change to the procedure reduced the anticipatory effect seen in the first experiment but did not induce the delayed awareness of action seen for the self and other-human actions. These findings indicate that intentions are attributed to others but not to machines. Interestingly, Wohlschläger et al. suggest that, in the machine action condition, modifying the sensory feedback in the form of a rubber hand *“may have activated to some extent a system for understanding biological actions”*.

The investigations above suggest that participants implicitly consider non-biological actions as distinct to self and other actions. This poses potential challenges in the development of such agents in order to facilitate optimal collaboration with humans. One such way to alter this may be the perceptual representation of the computer agent. Metrics used to measure the sense of agency, such as intentional binding offer opportunities to further test perceptual aspects of computer agents and their affect on the sense of agency during collaboration.

### Embodied agents

The perceptual representation of computer co-actors is a crucial consideration in HCI research. Within HCI there are two theoretical positions regarding this, the first holds that the “humanness” of the agent is key and that we feel fundamentally less connected to computer agents compared to other humans and avatars (Sheehan, 1991). The intentional binding studies outlined above (Wohlschläger et al., 2003; Obhi and Hall, 2011) feed into this human centric idea because they indicate that the emergence of “we-agency” is tightly linked to the nature of the “other” in joint action tasks. In addition, fMRI studies indicate that the brain regions activated during a collaborative task are more significantly activated by human partners compared to computer partners (Rilling et al., 2004). A contrasting perspective posits that humans automatically treat computers as social actors (Reeves and Nass, 1996); this is known as the Media Equation. This idea is based on the observation that humans orient socially to computers in the same manner as with other humans due to the fact that humans are “*very liberal in assigning humanity to an artificial stimulus as long as they have at least minimal human features and if they follow a social rule governing human-to-human interaction*” (Lee and Nass, 2003). The Media Equation goes further and suggests that designers of computer agents should focus on developing the cues that elicit the desired perceptions and responses from humans. There is empirical evidence to support this view. For example, the emotion conveyed in a computer agent’s tone of voice (happy or sad) affected the user’s perceived emotion of the content being conveyed (Nass et al., 2001).

These perspectives differ in the extent of the affect a biological resemblance of the computer agent has on our ability to attribute agency to the other. Typically these investigations explicitly assess attribution of agency through *post hoc* questionnaires (e.g., Nowak and Biocca, 2003). However, we have seen that explicit and implicit experiences of agency differ when co-acting with a computer partner (Obhi and Hall, 2011). Therefore, using implicit metrics such as intentional binding and sensory attenuation have the potential to yield important insights in this area of HCI research. Experimentally, computational techniques such as virtual reality offer promising new avenues to investigate the sense of agency in joint action by modifying and controlling the perceptual and motor requirements of tasks.

## Discussion and conclusion

This review has highlighted a new area of application for agency research-HCI. We have focused this review on a selection of opportunities to investigate the sense of agency in HCI settings; however this is certainly not intended to be an exhaustive list. Interaction techniques are evolving at a rapid rate. Once the validity and benefits of neurocognitive experimental techniques and implicit metrics such as intentional binding are established in HCI settings, they have the potential to inform the design of a wide range of new technologies. They will also provide valuable insights into how people experience interactions with technology and allow designers to more effectively tailor interaction experiences.

HCI is concerned with developing new or improving existing interfaces that sit between humans and computers. Thus this paper proposes that when developing novel interfaces or improving existing interfaces the user’s experience of agency is an important consideration. Whilst explicit measures, such as verbal report are currently utilized in HCI to assess agency, implicit measures are far less utilized. The relatively small number of prior studies that crossover between HCI and sense of agency research have been reviewed here. Of the studies reviewed, intentional binding has been the primary measure for implicit sense of agency. Intentional binding has been well replicated for tasks that require actions and outcomes on a sensorimotor (e.g., sub-second) timescale. Therefore, it is an ideal measure for assessing sense of agency in the many HCI tasks that involve agentive interactions at this timescale. However, we recognize that there are tasks in HCI that necessarily play out over longer timescales and for these tasks intentional binding may be less useful. Consider for example a computer game or robotic surgery in which individual actions combine to achieve a longer-term goal. Both the immediate experience of agency in individual actions and the user’s control over the longer-term goal will have an effect on the user experience. Further investigation will determine whether alternative measures, both implicit and explicit, may be better suited to HCI research on these kinds of scenarios.

We have also discussed both implicit and explicit computer assistance and the impact this has on a user’s sense of agency. For implicit assistance, research on agency can help to determine the extent to which users can be assisted whist still maintaining their experience of agency. For explicit assistance, the questions raised are different and surround the notion of how a system presents assistance to the user and how the user will attribute agency to an explicit computer co-actor. Sense of agency during joint-action is a current avenue of research, of which HCI techniques could provide assistance. Techniques such as virtual reality offer promising new avenues to investigate the sense of agency in joint action by modifying and controlling the perceptual and motor requirements of tasks. Within the context of virtual environments the notion of the virtual self is another interesting area for sense of agency research. It is commonplace for individuals to take actions and influence a virtual environment, by proxy, through a virtual representation of their self.

Ultimately we believe that HCI researchers can benefit from an increased understanding of underlying mechanisms involved in HCI tasks. Understanding these mechanisms will provide an additional evidence base for the design of interaction systems and this, in turn, may improve the efficiency of the design process and maximize the effectiveness of the end product. This understanding may prove particularly important for the growing body of HCI research focused on developing technology for groups whose sense of agency may differ from that of the normal population. This includes systems designed to support people with psychotic difficulties (Bickmore and Gruber, 2010; Gleeson et al., 2014) and Parkinson’s disease (Bächlin et al., 2010; Espay et al., 2010; Mazilu et al., 2014). We also think that HCI can benefit from new neurocognitive interventions that are being developed. For example, there is a burgeoning interest in cognitive enhancement through physiological interventions (such as non-invasive brain stimulation and psychopharmacology). This opens up new avenues for the HCI community, for example, by offering ways to artificially modify and improve user experience.

Finally, there is another dimension to investigating the sense of agency and HCI and that is one of personal responsibility in an ever-increasing society of technology users and intelligent machine interfaces. Situations where the distinction between computer and human controlled actions are blurred during computer assistance or joint action raise important legal and social questions for the sense of agency and responsibility. This is particularly so in safety critical scenarios. Consider for example self-driving cars, which automate the process of driving. One challenge in HCI is to develop optimal ways in which the interface can be presented to keep the distal feeling of control intact, but enable people to leave the proximal sensorimotor control to the machine. However a balance must also be struck such that the human “driver” retains a sufficient sense of responsibility to ensure the safe operation of the car. Understanding how the sense of agency is modified over time, when interacting with a semi-automated system, will also be important and may help to guide the recommended time spent using such interfaces. Within the HCI literature there are numerous examples of the consequences of poor interface design in safety critical situations, perhaps the most infamous of which was recorded in the partial nuclear meltdown at Three Mile Island. In this case conflicting information from a control panel, which operators had come to trust and rely on, contributed to initial operator inaction and delayed the response to the escalating crisis (Norman, 1988, pp. 43–44). Whilst the consequences will rarely be of such significance, the potential reduction in human responsibility as a consequence of increased interaction with intelligent interfaces is an important subject for further investigation. Research on HCI and agency will play a key role in shaping and informing decisions made in this area. The initial work of Berberian et al. (2012) showing operators’ diminished sense of agency in highly automated flight control scenarios offers a sense of the risk inherent in such situations.

## Conflict of interest statement

The authors declare that the research was conducted in the absence of any commercial or financial relationships that could be construed as a potential conflict of interest.
